# Antioxidant Potential of Crude Extract and Different Fractions of *Enhydra fluctuans *Lour

**Published:** 2010

**Authors:** Santanu Sannigrahi, Upal Kanti Mazuder, Dilip Kumar Pal, Sambit Parida, Sourabh Jain

**Affiliations:** a *St. Peter’s Institute of Pharmaceutical Sciences, Warangal, Andhra Pradesh -506001, India.*; b *Department of Pharmaceutical Technology, Jadavpur University, Kolkata-700 032, West Bengal, India.*; c *Department of Pharmaceutical Chemistry, Seemanta Institute of Pharmaceutical Sciences, Jharpokharia, Mayurbhanj-757 086, Orissa, India.*

**Keywords:** Antioxidant activity, *Enhydra fluctuans*, Different fractions, Free radical scavenging activity, Flavonoids

## Abstract

The antioxidant potential of crude methanol extract (CE) as well as chloroform (CF), ethyl acetate (EF) and n-butanol (NF) soluble fractions of *Enhydra fluctuans *Lour, which is widely used in indigenous system of medicine for different purposes, were studied. The antioxidant potential of extract/different fractions were evaluated using different in vitro antioxidant models. In addition, total amount of polyphenolics compounds, DPPH (1,1-diphenyl-2-picryl hydrazyl) radical, nitric oxide, superoxide anion, hydroxyl radical scavenging activities and reductive power of crude extracted its different fractions were determined. It was found that ethyl acetate fraction have maximum amount of polyphenolics compounds (179.7 ± 18.23 μg / mg pyrocatechol equivalent). This fraction was found more effective than crude extract and other fractions in all the above mentioned assays.

## Introduction

Free radicals, the partially reduced metabolites of oxygen and nitrogen, are highly toxic and reactive. Free radicals are linked with the majority of diseases like aging, atherosclerosis, cancer, diabetes, liver cirrhosis, cardiovascular disorders, etc. (1, 2). The most common reactive oxygen species are superoxide anion (O_2_^·-^), hydrogen peroxide (H_2_O_2_), peroxyl radical (ROO^·^) and highly reactive hydroxyl radical (OH^·^). The nitrogen derived free radicals are nitric oxide (NO) and peroxynitrite anion (ONOO^·^). Oxidation process is one of the most important route for producing free radicals in food, drugs and living systems. Antioxidants are the substances that when present in low concentration significantly delay or reduce the oxidation of the substrate (3). Antioxidants protect the body from damaging oxidation reactions by reacting with free radicals and other reactive oxygen species within the body and hindering the process of oxidation. So diseases linked with free radicals can be prevented by antioxidant therapy which gained an immense importance. 

Current research is now directed towards finding naturally occurring antioxidants particularly of plant origin. Currently available synthetic antioxidants like butylated hydroxy anisole (BHA), butylated hydroxy toluene (BHT), tertiary butylated hydroquinone and gallic acid esters have been suspected to cause negative health effects. Hence, strong restrictions have been placed on their application and there is a trend to substitute them with naturally occurring antioxidants. Moreover, these synthetic antioxidants also show low solubility and moderate antioxidant activity (4). BHA and BHT are suspected of being responsible for liver toxicity and carcinogenesis (5, 6). Traditionally used natural antioxidants from tea, wine, fruits, vegetables, spices, and medicine (e.g. rosemary and sage) are already exploited commercially either as antioxidant additives or a nutritional supplements (7). Also many other plant species have been investigated in search of novel antioxidants (8-11) but generally there is still a demand to find more information concerning the antioxidant potential of plant species. It has been mentioned that the antioxidant activity of plants might be due to their phenolic compounds (12). Flavonoids are a group of polyphenolic compounds with known properties which include free radical scavenging, inhibition of hydrolytic and oxidative enzymes and anti-inflammatory actions (13-15). The use of traditional medicine is widespread and plants still present a large source of natural antioxidants that might serve as leads for the development of novel drugs.

*Enhydra fluctuans *Lour. (Family: Compositae), an edible semi aquatic herbaceous vegetable plant with serrate leaves, grows all over India. The leaves are slightly bitter, cure inflammation, skin diseases and small pox (16). The leaves are also antibilious and are used in nervous diseases (17) and in torpidity of liver (18). It possesses nutritional value and its methanolic extract has been reported to have analgesic (19) and anti diarrhoeal activities (20). The leaves of *E. fluctuans *have been reported to have hypotensive activity (21). Chemical constituents like different sesquiterpene lactones were isolated from petroleum ether extract of *E. fluctuans *(22-25). Gibberelins (26) and cholesterol derivatives (27) have also been isolated from this plant.

Therefore, the objectives of present study were to determine the amount of total polyphenolic compounds and to evaluate the in vitro antioxidant activity of the crude methanol extract of *E. fluctuans *and its different fractions through different free radical scavenging assay. 

## Experimental

*Plant materials*

Fresh aerial part of the plant was collected in the month of December, 2006 and authenticated by Dr. H. J. Chowdhury, Joint Director, Botanical Survey of India, Howrah, West Bengal, India. The voucher specimen (SS/2007/01) has been deposited in our laboratory for further reference.

*Extraction and fractionation*

**Scheme 1:**
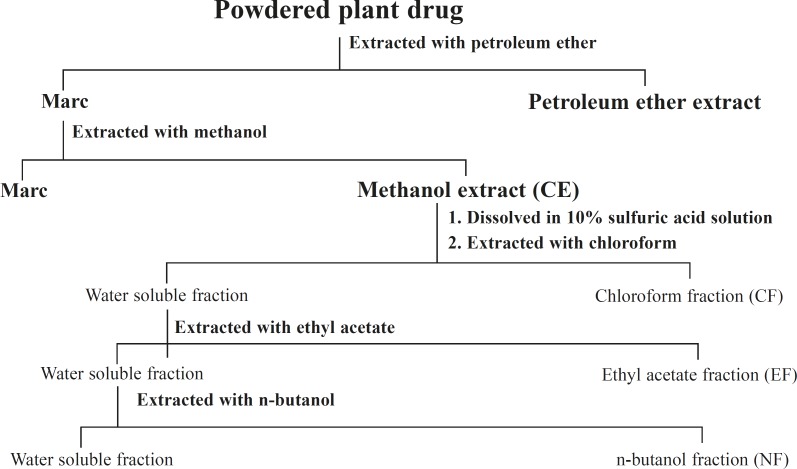
Scheme of extraction of aerial partsof *Enhydra fluctuans *Lour

The plant material was air dried until dryness at room temperature and then powdered with a mill. Air-dried and powdered aerial part (1.8 kg) was extracted successively with petroleum ether (60-80°C) and methanol using Soxhlet apparatus. The solvents were then removed under reduced pressure to obtained sticky residues. The crude methanolic extract (105 g), after removal of the solvent, was dissolved in 10% sulfuric acid solution and partitioned with chloroform, ethyl acetate and n-buatnol successively to give chloroform (5.5 g), EtOAc (12.9 g), n-BuOH (17.5 g) and water soluble fractions (scheme 1).

*Determination of total polyphenolic compounds*

The concentrations of phenolic content in all the fractions were determined with Folin–Ciocalteu’s phenol reagent (FCR) according to the method of Slinkard and Singleton (28). 1 mL of the sample solution (contains 1 mg) of the extract/fractions in methanol was added to 46 mL of distilled water and 1 mL of FCR, and mixed thoroughly. After 3 min, 3 mL of sodium carbonate (2%) were added to the mixture and shaken intermittently for 2 h at room temperature. The absorbance was measured at 760 nm. The concentration of phenolic compounds was calculated according to the following equation that was obtained from standard pyrocatechol graph (R^2^ = 0.9965):

Absorbance=0.001pyrocatechol(μg)+0.0033 

*DPPH radical scavenging activity*

The DPPH assay measured hydrogen atom (or one electron) donating activity and hence provided an evaluation of antioxidant activity due to free radical scavenging. DPPH, a purple-coloured stable free radical, was reduced into the yellow-coloured diphenylpicryl hydrazine which is measured spectrophotometrically at 517 nm (29). Briefly, 1 mL (0.1 mM) solution of DPPH in methanol was mixed with 3 mL of sample solution in water at different concentrations. Finally, after 30 min, the absorbance was measured at 517 nm. Decreasing of the DPPH solution absorbance indicates an increase of the DPPH radical-scavenging activity. IC_50_ values of the scavenging assay (concentration which can achieve 50% scavenging) are calculated by plotting the percentage of inhibition against the concentration to quantify the activity. DPPH radical-scavenging activity was calculated according to the following equation: 

% Inhibition = (A_0_-A_1_) / A_0_ × 100

where A_0_ was the absorbance of the control (without extract) and A_1_ was the absorbance in the presence of the extract.

*Nitric oxide scavenging activity*

Nitric oxide was generated from sodium nitroprusside, which at physiological pH liberates nitric acid. This nitric acid converts to nitrous acid and further forms nitrite ions (NO_2_^-^) which diazotize with sulphanilic acid and couple with naphthylethylenediamine (Griess reagent), producing pink color which can be measured at 546 nm (30). Sodium nitroprusside (10 mM, 2 mL) in phosphate buffer saline was incubated with the test compounds in different concentrations at room temperature for 30 min. Then, 0.5 mL of the incubated solution was added with 1 mL of Griess reagent and the absorbance was measured at 546 nm. The nitric oxide radicals scavenging activity was calculated according to the following equation: 

% Inhibition = (A_0_-A_1_) / A_0_ × 100

where A_0_ was the absorbance of the control (without extract) and A_1 _was the absorbance in the presence of the extract.

*Superoxide anion scavenging assay*

The scavenging activity of the crude extract and different fractions towards superoxide anion radicals was measured by the method of Nishimiki (31) with slight modification. Phenazine methosulfate-nicotinamide adenine dinucleotide (PMS-NADH) system was used for the generation of superoxide anion. Superoxide anion reduces nitro blue tetrazolium (NBT) into formazan at pH 7.8 at room temperature which can be determined by spectrophotometer at 560 nm. The decrease in absorbance in presence of extract/fractions indicates the consumption of superoxide anion by the tested compounds. About 1 mL of NBT (156 μM), 1 mL NADH (468 μM) in 100 mM phosphate buffer of pH 7.8 and 0.1 mL of sample solution of different concentrations were mixed. The reaction started by adding 100 μl PMS (60 μM). The reaction mixture was incubated at 25°C for 5 min and absorbance of the mixture was measured at 560 nm against blank samples. The percentage inhibition was determined by comparing the results of control and test samples.

*Hydroxyl radical scavenging activity*

The formation of hydroxyl radicals (OH·) from Fenton reagents was quantified using 2-deoxyribose oxidative degradation as described previously (32). Deoxyribose is degraded by OH· generated by Fenton systems and results in a series of reactions during which malondialdehyde (MDA) is formed which can be detected by its ability to react with thiobarbituric acid (TBA) to form a pink chromogen (33). The reaction mixture contained deoxyribose (2.8 mM); FeCl_3_ (100 mM); KH_2_PO_4_–KOH buffer (20 mM, pH 7.4); EDTA (100 mM); H_2_O_2_ (1.0 mM); ascorbic acid (100 mM), and various concentrations of the test compounds in a final volume of 1 mL solution. Ferric chloride and EDTA (when added) were premixed just before addition to the reaction mixture. After incubation of the reaction mixture at 37°C for 1 h, 1.0 mL of 2.8% trichloroacetic acid and 1.0 mL of 1% aqueous solution of TBA was added to the sample; test tubes were heated at 100°C for 20 min to develop the color. After cooling, TBARS formation was measured spectrophotometrically at 532 nm against an appropriate blank. The hydroxyl radical-scavenging activity was determined by comparing absorbance of the control with that of test compounds. 

*Reducing power assay*

The Fe^3+^ reducing power of crude extract and different fractions were determined by the method of Oyaizu (34). The extract (2.5 mL) at various concentrations was mixed with 2.5 mL of phosphate buffer (0.2 M, pH 6.6) and 2.5 mL of potassium ferricyanide [K_3_Fe(CN)_6_] (1%, w/v), followed by incubating at 50°C for 20 min. The reaction was stopped by adding 2.5 mL of trichloroacetic acid (TCA) solution (10%) and then centrifuged at 800 × g for 10 min. 2.5 mL of the supernatant was mixed with 2.5 mL of distilled water and 0.5 mL of ferric chloride solution (0.1%, w/v) and the absorbance was measured at 700 nm. Butylated hydroxyl toluene was used as reference standard. Higher absorbance of the reaction mixture indicated greater reducing power.

*Statistical analysis*

All data on all antioxidant activity tests are the average of triplicate analyses. The data were recorded as mean ±SD. The statistical significance of differences between groups was determined by analysis of variance (ANOVA), followed by Dunnett’s test for multiple comparisons among groups by using statistical software GraphPad Prism 4. Differences of P < 0.05 were considered statistically significant.

## Results and Discussion

There are different models available for evaluation of antioxidant activities. The chemical complexity of different extract and mixture of compounds present could lead to scattered results, depending on the test employed. Therefore, an approach with multiple assays for evaluating the antioxidant potential of extracts would be more informative and even necessary. In this study, different free radical scavenging activities was measured and all results were compared with standard antioxidant. 

*Total polyphenolics contents of the extracts*

Phenolic compounds are known as powerful chain breaking antioxidants. The concentration of phenolics in the extract/fraction expressed as μg of pyrocatechol per mg of the sample is shown in Table 1. Ethyl acetate fraction was found as the highest phenolics content fraction. The high concentration of polyphenolics in the ethyl acetate fraction may be due to purification and concentration of phenolics throughout the fractionation procedure and it is probably responsible for its high free radical scavenging activity. The FCR reducing capacity of different fractions is due to presence of hydroxyl groups present in the polyphenolics and flavonoids. The key role of phenolic compounds as scavengers for free radicals is emphasized in some previously published reports (35). It was reported that presence of hydroxyl groups contribute directly to antioxidant effect of the system and it also has an important role in preventing lipid oxidation (35).

**Table 1:** Total phenolic content of crude extract and fractions of *E. fluctuans*

**Sample**	**Phenolics as pyrocatechol equivalents (μg/mg)**
Crude extract (CE)	98.3 ± 8.69
Chloroform fraction (CF)	18.5 ± 3.11
Ethyl acetate fraction (EF)	179.7 ± 18.23
n-butanol fraction (NF)	27.5 ± 6.22

**Table 2:** IC_50_ value of extract/different fractions and standard antioxidants by different free radical scavenging methods

**Scavenging method **	**IC**_50_ **value (μg/mL) **				
	**EF**	**CE**	**NF**	**CF**	**Standard **
DPPH radical	23.4	66.5	62.7	>100	14.6 (Rutin)
Nitric oxide radical	26.1	74.5	60.5	>100	22.4 (Curcumin)
Superoxide radical	56.7	>100	94.8	>100	7.84 (Curcumin)
Hydroxy radical	23.5	>100	86.7	>100	5.85 (Catechin)

*DPPH radical scavenging activity*

**Figure 1:**
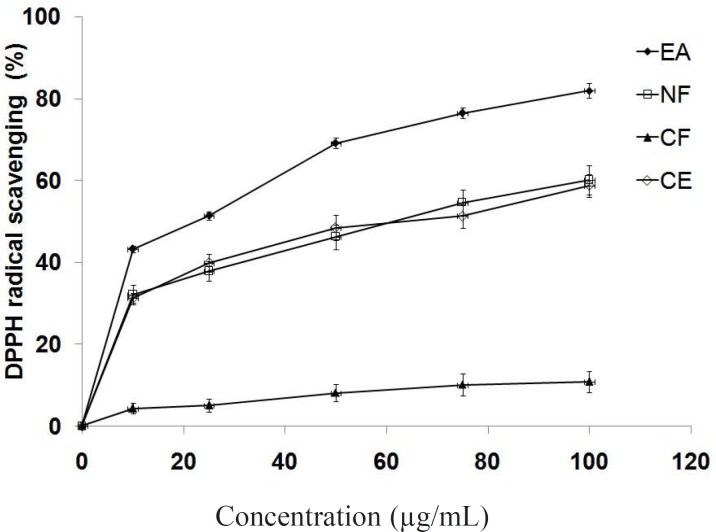
Radical scavenging potential of extract/fractions by DPPH method at different concentrations (μg/mL).

The results of the free radical scavenging potential of extract/different fractions tested by DPPH method are presented in Figure 1. Antioxidant reacts with DPPH, which is a nitrogen-centered radical with a characteristic absorption at 517 nm and converts it to 1,1-diphenyl-2-picryl hydrazine, due to its hydrogen accepting ability at a very rapid rate (36). The degree of discoloration indicates the scavenging potentials of the antioxidant. The ethyl acetate fraction showed highest DPPH radical scavenging activity (IC_50_ = 23.4 μg/mL) comparing to crude extract and other fractions. This assay provides information on the reactivity of different fractions with a stable free radical. The bleaching of DPPH absorption is representative of the capacity of tested compounds to scavenge free radicals independently from any enzymatic activity. The low IC_50_ value of ethyl acetate fraction is due to presence of high polyphenolics and flavonoids. 

*Nitric oxide scavenging assay*

**Figure 2:**
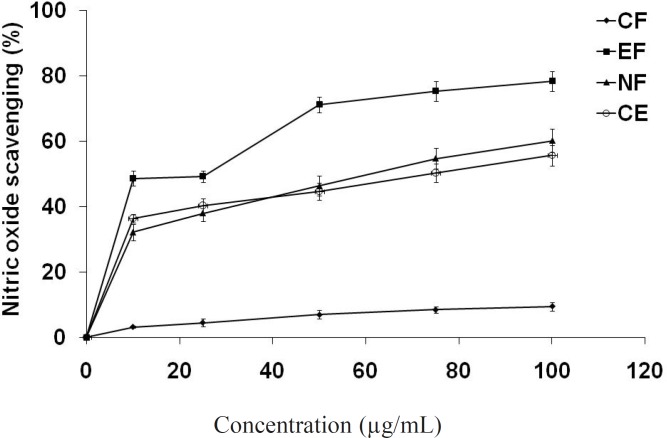
Nitric oxide scavenging potential of extract/fractions at different concentrations (μg/mL).

Different fractions of methanol extract of *E. fluctuans *also moderately inhibited nitric oxide in a dose dependent manner (Figure 2). Again, the highest nitric oxide scavenging activity was observed in ethyl acetate fraction (IC_50_ = 26.1 μg/mL) comparing to crude extract (IC_50_ = 74.5 μg/mL) and n-butanol fraction (IC_50_ = 60.5 μg/mL). Chloroform fraction showed very little effect at the concentration used in this study. Nitric oxide (NO) is a potent pleiotropic mediator of physiological processes such as smooth muscle relaxation, neuronal signaling, inhibition of platelet aggregation and regulation of cell mediated toxicity. It is a diffusible free radical which plays many roles as an effector molecule in diverse biological systems including vasodilation, neuronal messenger, and antimicrobial and antitumor activities. Studies in animal models have suggested a role for NO in the pathogenesis of inflammation and pain and NOS inhibitors have been shown to have beneficial effects on some aspects of the inflammation and tissue changes seen in models of inflammatory bowel disease (37). 

*Superoxide anion scavenging assay*

**Figure 3:**
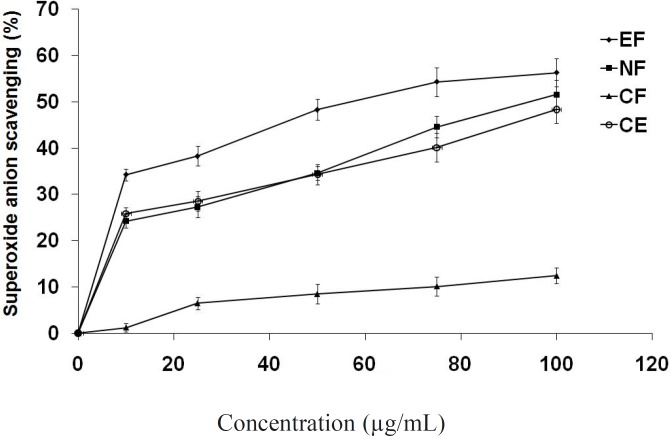
Scavenging potential of extract/fractions at different concentrations (μg/mL) on superoxide radicals generated by the PMS/NADH system

In the PMS/NADH-NBT system, superoxide anion is generated using a non-enzymatic reaction of phenazine methosulphate in the presence of NADH and molecular oxygen (38). It is well known that superoxide anions damage biomacromolecules directly or indirectly by forming H_2_O_2_, OH·, peroxylnitrite, or singlet oxygen during pathophysiologic events such as ischemic-reperfusion injury. The superoxide radical scavenging activities of active fractions were evaluated based on their ability to quench the superoxide radical generated from the PMS/NADH reaction. All the tested compounds showed superoxide anion quenching activity in a concentration dependent manner (Figure 3). Ethyl acetate fraction again showed maximum activity (IC_50_ = 56.7 μg/mL).

*Hydroxyl radical scavenging*

**Figure 4:**
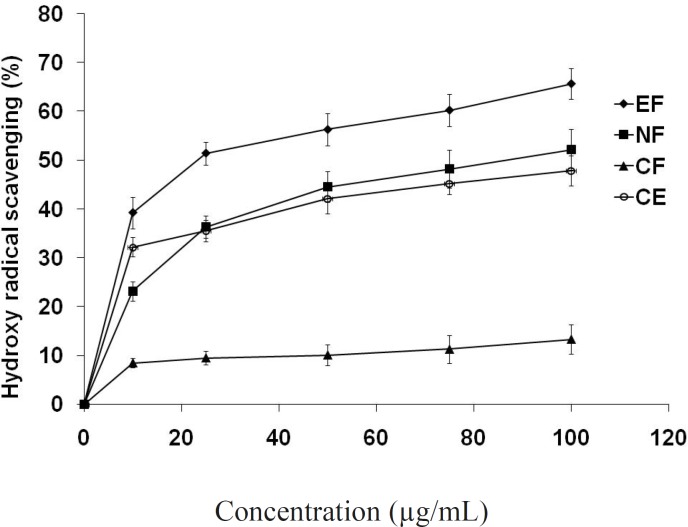
Hydroxy radical scavenging potential of extract/fractions at different concentrations (μg/mL) on deoxyribose degradation method

All the fractions suppressed hydroxyl radical mediated deoxyribose degradation in a concentration dependent manner (Figure 4). In this assay ethyl acetate fraction showed lowest IC_50_ value (23.5 μg/mL) comparing to n- butanol (IC_50_ = 86.7 μg/mL), crude extract and chloroform fraction. The hydroxyl radical is a highly potent oxidant that reacts with almost all biomolecules found in living cells when it reacts with polyunsaturated fatty acid moieties of cell membrane phospholipids, lipid hydroperoxides is produced (39). Lipid hydroperoxide can be decomposed alkoxyl and peroxyl radical and numerous carbonyl products such as malondialdehyde (MDA). The carbonyl products are responsible for DNA damage, generation of cancer and aging related diseases (40). Thus the decrease in the MDA concentration indicates the role of the tested compounds as an antioxidant. 

*Reducing power assay*

**Figure 5:**
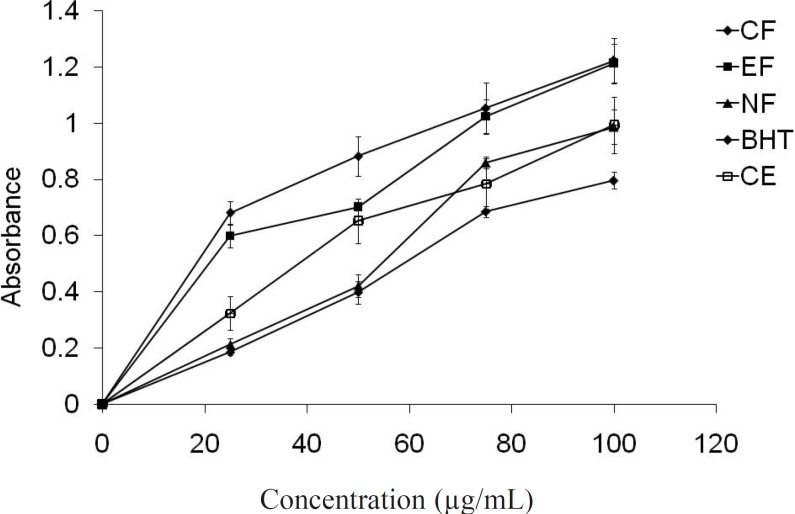
Reducing power of the extract/fractions by Fe^3+^ - Fe^2+^ transformation

The reducing power assay serves as a significant indicator of potential antioxidant activity. Although, different mechanism was proposed for the antioxidant activity such as prevention of chain initiation, binding of transition-metal ion catalysts, decomposition of peroxides, prevention of continued hydrogen abstraction, reductive capacity and radical scavenging (41). Crude extract and different fractions of *E. fluctuans *showed concentration-dependant reductive effects (Figure 5). The highest reducing activity was again observed for the ethyl acetate fraction. The reducing properties are generally associated with the presence of different reductants (42). The antioxidant action of reductants is based on the breaking of the free radical chain by donating a hydrogen atom. Reductones also react with certain precursors of peroxide, thus preventing peroxide formation. The reductive power of different fractions of *E. fluctuans *extract may be the reason for their antioxidant activity. 

## Conclusion

The results expressed in this study are the first information on the antioxidant activities of *E. fluctuans*. The crude extract and all the fractions showed free radical scavenging activity when tested in different models. Among all the fractions, the ethyl acetate fractions exhibited the highest free radical scavenging activity in all the tested models comparing to crude extract and n-butanol fraction. The crude extract was found to contain flavonoids, saponins and tannins. The highest scavenging activities of the ethyl acetate fraction can be ascribed to its flavonoids, which was concentrated in the fraction due to fractionation. The scavenging effect on DPPH and superoxide radicals represents the fraction direct radical scavenging activity. However, in the hydroxyl radical scavenging assay, hydroxyl radicals are generated by the Fenton reaction and the inhibition of deoxyribose degradation could be attributed to the inhibition of radicals. It is well documented that free radicals are responsible for several diseases. The present result confirms the free radical scavenging activity of the plant which can be accounted for the traditional uses of the plant in treating several diseases.
